# The Effects of Cholecalciferol Supplementation on Vitamin D Status Among a Diverse Population of Collegiate Basketball Athletes: A Quasi-Experimental Trial

**DOI:** 10.3390/nu12020370

**Published:** 2020-01-31

**Authors:** Nicole M. Sekel, Sina Gallo, Jennifer Fields, Andrew R. Jagim, Tammy Wagner, Margaret T. Jones

**Affiliations:** 1 Department of Nutrition and Food Studies, George Mason University, Fairfax, VA 22030, USA; nis123@pitt.edu (N.M.S.); twagner2@gmu.edu (T.W.); 2 Frank Pettrone Center for Sports Performance, George Mason University, Fairfax, VA 22030, USA; jfields8@gmu.edu (J.F.); mjones15@gmu.edu (M.T.J.); 3 School of Kinesiology, George Mason University, Manassas, VA 20110, USA; 4 Sport Medicine Research, Mayo Clinic Health Systems, Onalaska, WI 54650, USA; jagim.andrew@mayo.edu

**Keywords:** vitamin D, supplementation, 25-hydroxyvitamin D, collegiate athletes, basketball, skin pigmentation

## Abstract

Vitamin D may play a role in performance and injury risk, yet the required supplementation dosage for collegiate athletes is unclear. The objective of this study was to define the dosage of vitamin D_3_ supplementation required to beneficially affect serum 25-hydroxyvitamin D (25(OH)D) among a sample of collegiate basketball athletes. This was a quasi-experimental trial, participants were allocated to one of three groups of vitamin D_3_ daily at the beginning of pre-season training and dependent upon their baseline vitamin D status as follows: insufficient (<75 nmol/L) to 10,000 IU, sufficient (75–125 nmol/L) to 5000 IU and optimal (>125 nmol/L) to no supplementation. Follow-up assessments were completed ~ 5 months later in post season. The majority (*n* = 13) were allocated to 10,000 IU vs. *n* = 5 to 5000 IU and *n* = 2 to no supplementation. The 10,000 IU group showed the greatest change (35.0 ± 27.0 nmol/L) vs. the 5000 IU group (−9.3 ± 9.6 nmol/L) and no supplementation group (−41.6 ± 11.7 nmol/L, *p* < 0.01). Only 1 participant reached optimal status in the 10,000 IU group. In conclusion, a daily dosage of 10,000 IU vitamin D_3_ supplementation mitigated the high prevalence of vitamin D deficiency among collegiate basketball players but was insufficient for all to reach sufficient levels.

## 1. Introduction

Vitamin D is a fat-soluble micronutrient that occurs in two forms: vitamin D_2_ (ergocalciferol) and vitamin D_3_ (cholecalciferol). Research supports that vitamin D_3_ is more potent and exerts a longer duration of action physiologically than does D_2_, rendering it more efficacious in terms of beneficially affecting vitamin D status [1,2]. A recent systematic review and meta-analysis found that 56% of a total sample of 2000 athletes residing in 9 different countries including the United States had inadequate levels of vitamin D based on <80 nmol/L [3]. Examining vitamin D status among collegiate, indoor athletes is of particular relevance as research supports that not only are indoor athletes at greater risk [4,5] of suffering from insufficient vitamin D status due to limited sun exposure but young adults generally under consume a micronutrient-rich diet, proliferating risk factors of vitamin D deficiency [6,7]. The major source of vitamin D is provided through interaction of the skin; more specifically, the deep layer below the epidermis called the dermis—with ultraviolet beta (UVB) light. UVB light is a medium-wavelength, biologically active radiation sub-type with the ability to superficially penetrate the skin [8]. In the dermis, 7-dehydrocholesterol is converted into pre-vitamin D_3_ by UVB radiation. However, cutaneous pre-vitamin D3 synthesis and production is highly variable at different latitudes, times of the day, and seasons [9].

According to the National Academy of Medicine, several factors contribute to this disparity, including the skin’s ability to synthesize vitamin D efficiently including sunscreen use and high levels of skin melanin (dark pigmentation) [10]. Differences in skin pigmentation and thus dermal production of vitamin D may also contribute to this discrepancy as there is substantial evidence to support that synthesis of vitamin D in darker skin tones is lower when compared to lighter skin tones [11]. This is thought to be due to greater amounts of melanin in darker skin tones that less efficiently absorb UV wavelengths required to convert 7-dehydrocholesterol to vitamin D [11,12]. Secondly, to this protective nature, African Americans may require increased sun exposure than lighter skin tones in order to produce a similar amount of vitamin D [11,12]. Thus, darker skin tone athletes competing in indoor sports, with subsequent less exposure to sunlight, have been shown to be at risk for vitamin D deficiencies [13].

Studies examining vitamin D status in young, athletic populations are limited and highly underpowered [14]. Prior studies that have addressed this topic typically report data from non-athletic, older populations [15,16,17]. As natural dietary sources of vitamin D are limited, supplementation offers a safe, fiscal and efficacious method to combat insufficient status and return athletes to a healthy, sufficient state. Although, variable doses of vitamin D have been able to increase vitamin D status, the optimal dose has yet to be determined. The primary objectives of this pilot trial were to define and examine the prevalence of vitamin D deficiency among a diverse cohort of collegiate basketball athletes and secondly, to explore the appropriate dosage of vitamin D_3_ supplementation required to positively impact an athlete’s vitamin D status. Potentially confounding factors including adiposity, endogenous sources (i.e., sun exposure) and exogenous sources including both dietary and supplement intake were assessed. Findings from the current study will help inform whether the intervention dosages are appropriate to be tested in a more rigorous manner and on a larger scale.

## 2. Materials and Methods

### 2.1. Participants

The participants were National Collegiate Athletic Association Division I (NCAA-DI) men and women basketball athletes. Participation was voluntary and participants could autonomously withdraw from the study at any time and for any reason at no penalty or loss of benefits. Participants were required to be over the age of 18 years old and a healthy collegiate athlete belonging to one of the two aforementioned teams. The study was conducted in accordance with the Declaration of Helsinki, protocol approved by the George Mason University Institutional Review Board for Human Subjects (#978815-4) and all participants provided informed consent prior to participation in the study.

### 2.2. Study Design

This was an unblinded quasi-experimental trial with participants allocated to a vitamin D intervention regimen based upon their baseline circulating 25-hydroxyvitamin D (25(OH)D) status. Baseline assessments were conducted during the pre-competition season (in October 2018), and follow-up, post-season (between March and April 2019) and consisted of a blood draw, body composition assessment, anthropometric measures and questionnaires.

### 2.3. Vitamin D Intervention Regimen

The intervention regimen utilized in this trial was derived from the Academy of Nutrition and Dietetics’ Sports Nutrition Care Manual as well as, relevant athlete-specific literature and was implemented under the supervision of a Registered Dietitian/Sports Nutritionist [18,19,20]. At baseline, 25(OH)D status was determined and participants were allocated to one of three groups in an unblinded fashion based upon the supplementation regimen included in Table 1. The supplements contained 5000 IU/capsule and were formulated by NOW© foods, participants in the sufficient groups were advised to take 1 capsule daily and those in the insufficient group, 2 daily. Quality control testing was completed by Informed-Sport which tests for banned substances, certifies product purity and is one of three organizations recognized by the National Collegiate Athletic Association for approval of supplements to be distributed to collegiate athletes. The dosage was found to be within 10% of the target dosage. Participants were followed for ~ 5 months (length of the competition season), a sufficient time to see changes in vitamin D status as the half-life of 25(OH)D is ~ 15 days [21]. Certified athletic trainers designated to each team distributed supplements daily and watched participants take the supplement in order to ensure compliance.

### 2.4. Measurements

#### 2.4.1. Demographics

Demographic data were collected on age, race, nationality, basketball position, years of resistance training experience, years of basketball experience and current pregnancy status. Female participants were required to disclose pregnancy status as those with positive status were not permitted to participate in the study in order to mitigate unintended risk from the dual X-ray absorptiometry (DXA) scan. Race self-reported as African American, Mexican American/Latino, White/Anglo, Asian, Native American and other.

#### 2.4.2. Body Composition

DXA (Hologic, Horizon A model, Hologic Inc., Waltham, MA, USA) was used to assess body composition including body fat and lean mass. Additionally, bone area, bone mineral content (BMC) and areal bone mineral density (aBMD) of the whole body were measured. Participants were scanned using the whole-body scan mode (Hologic APEX software, ver. 5.5.3.1, Bedford, MA, USA). Calibration and procedures were performed to manufacturer specifications. The percent coefficient of variation (% CV) over the study period for the spine phantom (#26436) was 0.3% for BMD and for the whole-body phantom (#1104) was 1.5% for fat mass, 1.5% for lean mass, 0.1% for total mass and 1.7% for % body fat. All tests were performed under the supervision of a trained technician.

#### 2.4.3. Skin Pigmentation

Skin pigmentation was measured at baseline via a portable, computerized spectrophometer (CM-600D, Konica Minolta, Ramsey, NJ, USA). This measurement was performed on each participant’s upper underarm. Athletes were classified into 5 skin phototypes: dark (≤10°), olive (10–28°), medium (28–41°), fair (41–55°) and very fair (>55°). However, based upon small numbers of participants in each group, these groups were reclassified as: dark-olive (≤10–28°), medium (28–41°) and fair-very fair (41–55°<) [22,23].

#### 2.4.4. Exogenous Intake of Vitamin D

In order to assess vitamin D intake from dietary sources, between 1 and 3, 24-hour recalls were conducted by a Registered Dietitian via phone call and in-person with each participant. If more than 1 recall was conducted, results pertaining to the same participant were averaged to assess usual intake. The Nutrition Data System for Research (NDSR) was utilized to quantify the total vitamin D (IU/day) intake based upon the 24-hour recall results. NDSR’s Dietary Supplement Assessment Module (DSAM) captures information pertinent to supplements [24]. NDSR collects information via the United States Department of Agriculture (USDA) databases, product labels, scientific literature, foreign food composition tables, and National Health and Nutrition Examination Survey (NHANES) (2013–2014) Dietary Screener Questionnaires (DSQ) Database and supplements added by the Nutrition Coordinating Center (NCC), an establishment of the National Institutes of Health (NIH) [24]. Missing foods or supplements typically utilized by the participants were added to the NDSR database prior to analysis.

#### 2.4.5. Endogenous Intake of Vitamin D

During the study visit, a sun exposure questionnaire was utilized to assess sun exposure, winter travel and sunscreen use. Sun exposure data were collected including recent (within the past 3 months), travel to a warmer climate, duration of stay in a warmer climate, hours of direct sunlight, body part most exposed to direct sunlight, sunscreen usage, frequency of application and application site, time spent outdoors, residence over the winter and summer months, and Sun Protection Factor (SPF) brand and frequency of usage.

### 2.5. Outcome Assessment: Serum 25(OH)D Concentrations

Upon arrival to the laboratory, participants were seated in an upright position and a blood sample was collected from an antecubital vein using standard sterile phlebotomy procedures. Blood was drawn into a 5 ml vacutainer tube that contained no additive (BD Biosciences, San Jose, CA, USA). Samples were allowed to coagulate in cooling beds for ~30 minutes, and subsequently centrifuged at 2500 RPM for 15 minutes (Eppendorf 5702R, Eppendorf North America, Hauppauge, NY, USA). After centrifugation, the serum was stored at −80 °C until batch analysis. The serum concentration of 25(OH)D was measured in duplicate using a commercially available ELISA kit (Monobind, Lake Forest, CA, USA) and a plate reader (Epoch, BioTek, Winooski, VT, USA). The intra-assay coefficient of variation for 25(OH)D was 4.5%. The following cutoffs were used to determine 25(OH)D (nmol/L) status based on current literature [6,9,25]: ≤75 = insufficient; 75–125 = sufficient; ≥125 = optimal.

### 2.6. Statistical Analysis

SPSS Version 24.0 (IBM, Armonk, NY, USA) was used for data analysis. The Shapiro–Wilks test was used to test normality of all variables. Mean ± SD were used to describe continuous and *n* (%) for categorical variables. A one-way analysis of variance (ANOVA) or chi square (*X*^2^) test were used to assess mean differences in characteristics across intervention groups. ANOVA was used to assess the change in 25(OH)D from baseline to follow-up and *X*^2^ to assess differences in vitamin D status at follow-up across intervention groups. A Bonferroni post-hoc test was performed to assess for differences among groups. A Spearman’s correlation was performed to assess for correlations between the change in 25(OH)D and baseline 25(OH)D as well as body composition indices.

## 3. Results

Table 2 includes participant characteristics overall and by intervention group. The mean age was 20.25 ± 0.85 years old, 12 (60.0%) self-reported as African American and 10 (50.0%) were female. The majority of participants (*n* = 13) were allocated to the high-dose supplementation group (10,000 IU daily) vs. *n* = 5 allocated to 5000 IU daily and *n* = 2 to no supplementation. Overall, 10 (76.9%) participants allocated to the high dose supplementation group (10,000 IU daily) were male and 11 (84.6%) African American and similarly 10 (90.91%) were dark or olive skin tone (*p* < 0.05). Differences among groups were noted for whole body baseline BMD Z-score (*p* = 0.027) and lean body mass (*p* = 0.004).

Table 3 shows no statistically significant differences in vitamin D status at follow-up (*p* = 0.395). In the non-supplemented group, one athlete remained at optimal status while the other athlete fell to sufficient status. Among the 5000 IU daily group, 3 (75%) participants remained at sufficient status while 1 athlete (25%) fell to insufficient status at follow-up. Among the high dose intervention group (10,000 IU daily), 3 (23%) remained insufficient, 9 (69%) achieved sufficient status, 1 (8%) attained optimal status.

**Figure 1:**
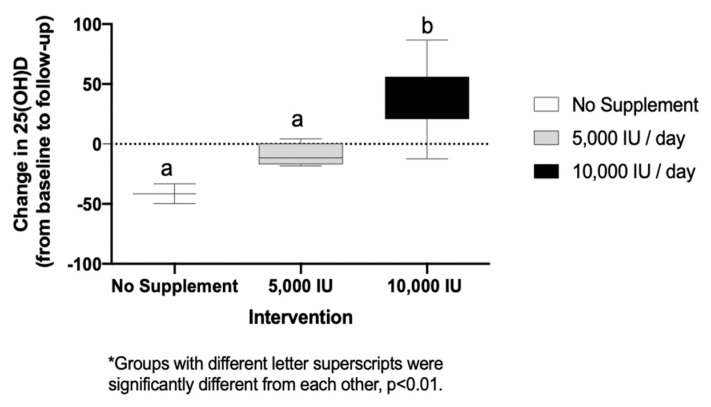
Change of 25(OH)D concentrations (nmol/L) by intervention group.

Figure 1 displays the change of 25(OH)D D concentrations from baseline to follow-up by intervention group. There was a statistically significant increase in 25(OH)D between the 10,000 IU group (+35.0 ± 27.0 nmol/L) as compared to both the non-supplemented group (−41.6 ± 11.7 nmol/L) and the 5000 IU group (−9.3 ± 9.6 nmol/L, *p* = 0.001). A statistically significant correlation was observed between the change in 25(OH)D (from baseline to follow) and four the body composition indices as follows, fat mass (*r*_s_ = −0.65, *p* = 0.01), lean body mass (LBM) (*r*_s_ = 0.53, *p* = 0.05), LBM percentage (*r*_s_ = 0.83, *p* = 0.01) and body fat percentage (*r*_s_ = −0.80, *p* = 0.01). Additionally, there was a significant correlation between change in 25(OH)D and baseline 25(OH)D (*r*_s_ = −0.78, *p* = 0.01).

**Figure 2:**
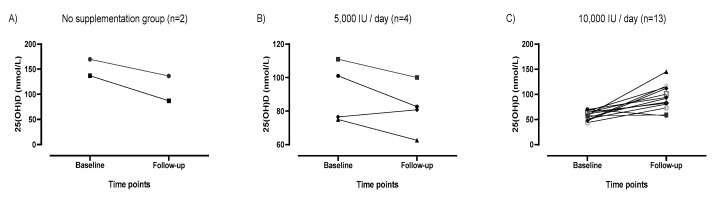
25(OH)D concentrations at baseline and follow-up by group and individual.

Figure 2 displays the aforementioned difference of 25(OH)D concentrations at baseline and follow-up by individual and group (panels A–C). Panels A and B show the majority of participants allocated to no supplementation or 5000 IU daily decreased serum 25(OH)D over the course of the trial. Panel C shows only 1 of the 13 participants allocated to 10,000 IU daily decreased serum 25(OH)D, the remaining 12 participants (92%) all increased their serum 25(OH)D.

## 4. Discussion

Overall, 13 of the 20 (65%) participants were vitamin D insufficient at baseline (based upon 25(OH)D of <75 nmol/L). This result is consistent with a recent systematic review and meta-analysis wherein 56% of a total sample of 2000 athletes residing in 9 different countries including the US had inadequate levels of vitamin D (based upon <80 nmol/L) [3]. Albeit a pilot study, the current results provide further evidence of the high prevalence of vitamin D insufficiency among a sample of highly-trained, NCAA-DI basketball athletes. It is well documented that limited sun exposure, latitude of residency and seasonal variations may inhibit subcutaneous synthesis of vitamin D [4,9,13]. Baseline tests were performed in October, and due to the half-life of vitamin D_3_, were indicative of the participants’ vitamin D status during the summer months [26]. Hence, the decreased 25(OH)D concentrations observed among the no supplementation and 5000 IU/day groups were likely reflective of a seasonal decline. The 10,000 IU daily was the only dosage which appeared to be protective against this decline in 25(OH)D concentrations among participants (Figure 2). Further, most basketball athletes in the current study were of darker skin pigmentation and trained exclusively indoors, both of which further reduce dermal production of vitamin D_3_ and predispose participants to vitamin D deficiency [11,13,27]. Darker-skinned athletes, 10 (90.9%) among our sample, exhibited heightened risk of vitamin D insufficiency at baseline, and none of the participants with fair or very fair skin fell into the insufficient category at baseline.

Existing research largely endorses supplementation efficacy. Backx et al. [28] examined vitamin D deficiency from elite Dutch athletes over the course of one year and supplemented based upon their degree of insufficiency at baseline at 400, 1100 or 2200 IU vitamin D_3_ daily. Conclusively, serum 25(OH)D concentration increased more in the 2200 IU/day group (+50 ± 27 nmol/L) than the sufficient group receiving no supplements (+4 ± 17 nmol/L; *p* < 0.01) [28]. Cumulatively, the 2200 IU/d dosage resulted in a sufficient 25(OH)D concentration in 80% of the athletes over the duration of 1 year. This was the result after 70% of those athletes were categorized as insufficient or deficient at baseline based upon the aforementioned defined intervals [28]. Similarly, Close et al. [29] examined the vitamin D concentrations in non-supplemented, UK-based, male professional athletes over an 8-week duration during the winter months. The intervention group received a daily supplement over the duration of 8 weeks of 5000 IU of vitamin D_3_, whereas the control group received an inert placebo. As a result of the intervention, serum total 25(OH)D concentration significantly increased serum total 25(OH)D from baseline (29 ± 25 to 103 ± 25 nmol, *p* < 0.01), whereas the placebo showed no change (53 ± 29 to 74 ± 24 nmol, *p* = 0.12) [29]. Following supplementation, however, 60% of the vitamin D supplemented group had vitamin D concentrations greater than 100 nmol/L and could therefore be classified as having reached optimal status [29]. Despite the results of prior literature and the usage of a relatively large dose of vitamin D_3_, of 10,000 IU per day in the current study, the majority of participants were unprotected against the observed decline in 25(OH)D concentrations. Potential mediating factors may include increased physical activity, increased physiological stress and increased metabolic requirements necessitated by highly trained athletic populations such as NCAA-DI athletes. Aforementioned factors that predispose to vitamin D deficiency include increased adiposity [25,30,31,32], skin tone [11,12], limited cutaneous synthesis dependent upon limited sun exposure, residency latitude and seasonal variations [9].

According to the National Academy of Medicine, the Recommended Dietary Allowances (RDA) of vitamin D for a healthy North American population is 600 IU [21]. This recommendation does not target athletes, which may have increased needs, nor does it account for those who are vitamin D deficient. Results from prior research indicate dietary vitamin D intake was higher amongst participants than athletes [27,33], which is likely due to use of food supplements fortified with vitamin D. Previous research suggests a wide range of vitamin D supplementation with as high as a single dose of 300,000 IU [10,34]; however, there is currently a lack of consensus regarding the ideal dose and it may largely depend on baseline values and the presence of any previously mentioned confounding factors. Vitamin D is a fat-soluble vitamin, hence, there is a potential risk of toxicity and caution must be exerted when establishing supplementation recommendations. It should be noted that prior research has found evidence to support risk associated with this level of supplementation [35]. However, in the current study, a daily dosage of 10,000 IU of vitamin D_3_ led to increases in 25(OH)D concentrations (+35.1 nmol/L), while a dosage of 5000 IU daily led to a mean decrease (−9.34 nmol/L). In addition, only 1 of the 13 (8%) allocated to the 10,000 IU group achieved optimal status, 9 participants of 13 (69%) achieved sufficient status after the duration of ~5 months. The most efficacious dosage to impact an individual’s status beneficially is difficult to ascertain based upon difference in skin pigmentation, level of adiposity, season and baseline vitamin D status. Further optimal status as defined as serum 25(OH)D concentrations >125 nmol/L were difficult to achieve and maintain, as only two participants in our sample were able to achieve optimal status at follow-up. Our current results suggest 10,000 IU daily was more efficacious in preventing declines in 25(OH)D as observed among those in the 5000 IU daily or no supplementation groups; however, specific guidelines for elite collegiate basketball athletes are in need of development.

Current results indicate a positive association between baseline 25(OH)D concentrations and bone mineral density (*p* = 0.029) and a negative association between 25(OH)D and lean body mass (*p* = 0.004). Prior results with male athletes support a positive relationship between physical activity, lean body mass and bone mineral density [36]. This inconsistency may be due to the high degree of leanness in the current sample, particularly among male participants. Current average values for body fat percentage for college age men is approximately 15% [37]. In the current sample, the male participants exhibited an average percent fat of 13.5%. Current results also indicate an inverse association between the change in 25(OH)D observed with baseline 25(OH)D status, fat mass and percentage body fat (*p* = 0.01). Hence, higher body fat and fat mass were associated with a lower change in 25(OH)D in response to the intervention. Vitamin D status as it relates to body composition largely originated in scientific literature with the fat sequestration hypothesis. This was first identified as a significant correlation between white, obese participants and low circulating serum 25(OH)D [31]. This was supported in the athletic population by Heller et al. [32] who identified that larger athletes with corresponding excess adiposity may be at higher risk for both vitamin D insufficiency and deficiency, even after controlling for sex in a mixed model. Hidelbrand et al. [13] also published similar findings suggesting that athletes with body composition in the overweight or obese category had lower serum 25(OH)D (*p* < 0.05) compared with those who were normal or below recommended fat percentages. Among participants allocated to the 10,000 IU group, 3 remained categorically insufficient at follow-up, but only 1 of these participants decreased in serum 25(OH)D from baseline to follow-up. All three of these participants exhibited higher fat mass compared to the rest of the sample. These results were: 15.44 kg for the male participant (male fat mass mean = 11.7 kg) and between 29.6 and 42.5 kg for the two female participants (female fat mass mean = 21.2 kg). This result suggests that the lack response in 25(OH)D, particularly among the 10,000 IU supplemented group, may have been inhibited due to increased adiposity. Among post-menopausal women, Gimigliano et al. [38] found hypovitaminosis D and overweight may negatively affect muscle mass and function hence, suggesting additional detriments.

### Strengths and Limitations

The current study was a quasi-experimental trial with treatment dosages based upon clinical practice guidelines in conjunction with a Registered Dietitian. Further, participants were allocated to one of three intervention groups based upon baseline status of 25(OH)D concentration, which has not been the case in the majority of previous studies. This discrepancy in relevant research is exemplified in Heaney et al. [39] who in conjunction with results from a meta-analysis performed by Bischoff-Ferrari et al. [15] states that among over 30,000 participants included in randomized controlled trials pertinent to vitamin D status, baseline 25(OH)D concentrations were available for only 14%. The other trials supplemented participants based upon a standard dosage. As baseline status will affect response, failing to assess an individual’s baseline status and subsequently issuing a standard dosage may not be efficacious [15,39]. Additionally, results from the current study contribute to an emerging pool of literature pertinent to American, indoor, elite collegiate athletes of diverse skin-tones, sex and adiposities. The primary limitations were the small sample size of 20 participants, limiting the results’ acceptability and generalizability and recall bias necessitated by dietary, supplement and sun exposure recall. Compliance represented another limitation, potentially affected by frequent team travel. Further, it should be noted that different assay systems can produce slightly different values. In this study, ELISA methods were used to determine vitamin D concentrations. While this assay has been validated against clinically accepted immunoassays, it is possible that this method can provide for variability in concentrations. Prior research indicates a positive association between serum 25(OH)D concentrations and daily dietary vitamin D intake [9]. Yet, high variability as a result of self-disclosure for these measures among our sample may have affected results.

## 5. Conclusions

There was a high prevalence of vitamin D insufficiency among, predominately African-American, elite collegiate basketball athletes. Supplementation as high as 10,000 IU daily was unable to achieve sufficient status among all participants although it appears to be protective against seasonal declines in 25(OH)D concentrations. Conversely, a dosage of 5000 IU daily was insufficient and failed to attenuate against seasonal decline. High adiposity and the lack of ability to achieve a categorically optimal concentration of 25(OH)D above 125 nmol/L may help explain the results. Research, with a larger sample, is warranted to aid in the development of screening protocols which will enable medical and sports nutrition staff to identify key risk factors of hypovitaminosis D.

## Figures and Tables

**Table 1:** Vitamin D supplementation regimens.

Vitamin D Status Definition	Baseline 25(OH)D Concentration	Supplementation Regimen (IU/d) ^1^
Insufficient	<75nmol/L (30 ng/mL)	10,000 IU/cap/day
Sufficient	75–125 nmol/L (30–50 ng/mL)	5000 IU/cap/day
Optimal	>125 nmol/L (50 ng/mL)	No Supplementation

^1^ Treatment dosages based on clinical practice guidelines in conjunction with a Registered Dietitian [18,19,20].

**Table 2:** Baseline characteristics of participants by intervention groups. Presented as mean ± SD for continuous variables and *n* (%) for categorical variables.

Variable	Overall (*n* = 20)	Intervention Groups			*p* Value ^7–9^
		No Supplement (*n* = 2) ^1^	5000 IU/day (*n* = 5) ^2^	10,000 IU/day (*n* = 13) ^3^	
Serum 25(OH)D (nmol/mL)	75.56 ± 31.95	153.38 ± 23.16 ^a^	89.4 ± 15.89 ^a^	58.27 ± 8.62 ^a^	<0.001
Age (years)	20.25 ± 0.9	21 ± 0.0	20.6 ± 0.9	20 ± 0.8	0.175
**Sex**					
Male	10 (50%)	0 (0%)	0 (0%)	10 (76.9%)	0.005
Female	10 (50%)	2 (20%)	5 (50%)	3 (30%)	
**Self-reported race**					
White/Anglo	6 (30.0%)	2 (100%)	2 (40%)	2 (15.4%)	0.027
African American	12 (60%)	0 (0%)	1 (20%)	11 (84.6%)	
Latino	1 (5%)	0 (0%)	1 (20%)	0 (0%)	
Mixed	1 (5%)	0 (0%)	1 (20%)	0 (0%)	
**Skin pigmentation** **(Inner, upper arm)**					
Dark or olive (≤10–28°)	12 (70.6%)	0 (0%)	2 (50%)	10 (90.9%)	0.022
Medium (28–41°)	4 (23.5%)	2 (100%)	1 (25%)	1 (9.1%)	
Fair or very fair (41°<)	1 (5.9%)	0 (0%)	1 (25%)	0 (0%)	
**Body Composition** ^4^					
Whole body BMD (g/cm)	1.28 ± 0.1	1.21 ± 0.0	1.26 ± 0.0	1.3 ± 0.1	0.447
Whole body BMD Z-Score	1.1 ± 0.8	1.6 ± 0.0 ^a^	1.78 ± 0.7 ^a,b^	0.76 ± 0.7 ^a,c^	0.029
Fat Mass (kg)	16.45± 8.2	16.62 ± 6.5	18.23± 4.5	15.73± 9.7	0.859
Lean Mass (kg)	63.82± 11.3	54.25 ± 4.9 ^a^	53.1 ± 5.2 ^a,b^	69.44± 9.6 ^a,c^	0.004
Lean mass (kg)/total mass (kg) × 100 (%)	76.8 ± 6.7	74.04 ± 4.9	71.88 ± 3.0	79.12 ± 7.0	0.094
Body Fat (%)	19.45 ± 7.1	22.2 ± 5.3	24.4 ± 3.3	17.13 ± 7.5	0.124
**Dietary intake** ^5^					
Vitamin D, total (IU/day)	350.02 ± 333.0	367.07 ± 211.4	359.41 ± 304.1	343.78 ± 375.3	0.994
**Sun exposure** ^6^					
Time spent outdoors (weekday), <40 min.	17 (85%)	2 (100%)	4 (80%)	11 (84.6%)	0.798
Time spent outdoors (weekend), <40 min.	12 (60%)	1 (50%)	1 (20%)	10 (76.9%)	0.083
Average minutes/day of direct sunlight exposure, <30 min.	15 (75%)	1 (50%)	4 (80%)	10 (76.9%)	0.684

^1^ Participants allocated to group at baseline if fell within optimal range (>125 nmol/L); ^2^ Participants allocated to group at baseline if fell within sufficient range (75–125 nmol/L); ^3^ Participants allocated to group at baseline if fell within insufficient range (<75 nmol/L); ^4^ Based on dual-energy x-ray absorptiometry (DXA); ^5^ Based on Nutrition Data Systems for Research (NDSR) data at follow-up; ^6^ Self-reported at baseline; ^7^ A one-way analysis of variance (ANOVA) or chi square (X^2^) test were used to assess mean differences in characteristics across intervention groups; ^8^ Different letter superscripts identify significant differences among groups as per post-hoc testing; ^9^ *p* value of ≤0.05 determines statistical significance.

**Table 3:** 25(OH)D status at follow-up by intervention dosage. Presented as *n* (%).

Status at Follow-Up	Status at Baseline			
	No Supplement (*n* = 2)	5000 IU/day (*n* = 5)	10,000 IU/day (*n* = 13)	*p* Value ^2–3^
Insufficient < 75 nmol/L	0 (0%)	1 (25%)	3 (23.1%)	0.395
Sufficient 75–125 nmol/L	1 (50%)	3 (75%)	9 (69.2%)	
Optimal > 125 nmol/L	1 (50%)	0 (0%)	1 (7.7%)	

^1^ Total of 5 participants were allocated to 5000 IU D_3_ at baseline but only 4 remained at follow-up due attrition; ^2^ Chi square (X^2^) test were used to assess differences in vitamin D status at follow-up across intervention groups; ^3^ *p* value of ≤0.05 determines statistical significance.
